# Mediation Effect of Age Category on the Relationship between Body Composition and the Physical Fitness Profile in Youth Handball Players

**DOI:** 10.3390/ijerph17072350

**Published:** 2020-03-31

**Authors:** Jorge Molina-López, Iván Barea Zarzuela, Jesús Sáez-Padilla, Inmaculada Tornero-Quiñones, Elena Planells

**Affiliations:** 1 Faculty of Education, Psychology and Sport Sciences, University of Huelva, Avda. Tres de Marzo, s/n, 21071 Huelva, Spain; ivan.barea121@alu.uhu.es (I.B.Z.); jesus.saez@dempc.uhu.es (J.S.-P.); inmaculada.tornero@dempc.uhu.es (I.T.-Q.); 2 Institute of Nutrition and Food Technology, Biomedical Research Center, Health Sciences Technological Park, University of Granada, 18010 Granada, Spain; elenamp@ugr.es; 3 Faculty of Pharmacy, University of Granada, Campus de Cartuja, s/n, 18071 Granada, Spain

**Keywords:** body composition, physical fitness, physiological profile, handball youth, mediation

## Abstract

The aims of the present study were (1) to determine the differences in body composition and the physical and physiological profile of handball youths across age categories, and (2) to analyze the mediation effect of different categories on the relationship between lean mass or fat mass and specific physical handball capacities. Fifty-four young handball players aged 13 to 18 were assigned to U14 (13- and 14-year-olds), U16 (15- and 16-year-olds), and U18 (17- and 18-year-olds). Body composition was measured using multi-frequency bioelectrical impedance (Inbody®, 230). Handball physical fitness was assessed using handgrip force, jumping tests (squat jump, countermovement jump, countermovement jump with aimed arms), a 30-m sprint, a change-of-direction test (T-half agility test and modified Illinois test), and a Yo-Yo intermittent recovery test level 1. Simple mediation analysis was performed to analyze whether the category mediated the relationship between lean mass or fat mass and physical capacities. No significant differences were observed according to category for the majority of the measured parameters, except for height, lean body mass, and arm span. Lean body mass increased significantly as player category increased (*p* < 0.05; **∆** = 4.66–9.38; effect size (ES) = 0.96–1.92). The increase in handball category enhanced the majority of the physical capacities evaluated; however, these differences were reduced between the U16 vs. U18 categories. The indirect mediation effect suggests that handball category mediated the relationship between lean mass and upper and lower strength, velocity, agility, and cardiorespiratory fitness. In contrast, an indirect effect suggests that category mediated the relationship between fat mass only in agility and cardiorespiratory fitness. We conclude that U18s showed better body composition parameters, as well as better physical performance scores. Handball category clearly mediated the relationship between body composition through lean mass and fat mass and the physiological profile in handball youth, but lean mass proved to be more relevant when mediating physical performance.

## 1. Introduction

Researchers in sport performance and talent identification are becoming increasingly focused on the study of sport-specific characteristics and requirements [1]. Handball is characterized by requiring repeated accelerations, sprints, jumps, rapid changes of direction, a large number of throws, and frequent body contact between players [2,3,4]. In order to be successful, regular actions such as feints, shots, and entrances have to be carried out with maximum intensity [5]. In handball youths, annual age-grouping is a widely used strategy to divide athletes into categories based on their date of birth. Therefore, depending on the age category, handball performance could be influenced by the evolution of age itself, of body composition, and the evolution of technical skills, tactical understanding, and physical abilities [6].

To date, different studies have evaluated different qualities considered to be decisive in handball youths, such as strength, power, speed, and agility, as well as high-intensity intermittent aerobic training [6,7,8,9,10]. In this line, previous studies were focused on the influence of biological maturation, anthropometry, and physical performance on different game positions [6,8,9,11,12], as well as their evolution in elite and non-elite handball youths throughout a sports season [13]. A recent study [6] underlined the progressive age-related development of factors influencing performance throughout adolescence, indicating the importance of age-categorized competition in handball until at least the age of 19. In addition, elite and non-elite players were shown to be controlled by maturity status, and large positive changes were proven in anthropometry and physical performance (strength and speed), especially in the younger age categories [9]. The lack of significant effects reveals that elite players did not improve their physical performance level more rapidly during the three years of this study than the non-elite players [13]. At present, there are still some concerns regarding the physical performance of young handball players and how body composition, namely, lean mass and fat mass, may differently enhance physical performance, especially from the beginning of their sports specialization stage.

As mentioned above, anthropometric characteristics have been shown to be decisive in youth and adult team handball [6,12,14]. Traditionally, body mass index (BMI) and indirect fat mass calculation are used to assess body composition in handball youths, but due to the limitations of this parameter (i.e., BMI correlates not only with fat mass but also with lean mass; moreover, BMI gives no indication of body fat distribution), in recent years, there has been a need for more precise techniques [15]. Although the most recent research [6,7] showed a relationship between the performance of both upper and lower limb strength and the majority of the anthropometric characteristics of male handball players (body mass, height, or indirect fat percentage calculation), there are no studies that analyze the different body compartments with more precise techniques, such as multi-frequency bioelectrical impedance analysis, and their influence on each one of the analyzed abilities. Therefore, a more comprehensive understanding about lean mass and fat mass predominance from the beginning of handball specialization is necessary, determining the mediation effect of age category on the relationship between body composition and physical performance.

The present investigation aimed (1) to determine the differences in body composition (lean body mass or fat mass) and the physical and physiological profile of handball youths across age categories, and (2) to analyze the mediation effect of different categories on the relationship between lean mass or fat mass and specific physical handball capacities. Our initial hypotheses were that body composition and physical performance would develop over the age categories, and that the age category would mediate differently the relationship between lean mass and fat mass and the performance-determining physical capacities in young handball players.

## 2. Materials and Methods

### 2.1. Subjects

The present study enrolled a total of 54 male handball players aged 13–18 from Pedro Alonso Niño Handball Club located in the Huelva region. The 54 players were divided into three different categories by birth year: the youngest group was born in 2005–2006 and played in the 13–14-year-old category (U14), the second group was born in 2003–2004 and played in the 15–16-year-old category (U16), and the oldest group was born in 2001–2002 and played in the 17–18-year-old category (U18). Handball players were matched and competed by chronological age according to the Handball Federation rules. All players were in good health and passed a medical examination before starting their handball season. Measurements were taken during the competitive phase of the handball season. During this period, the training volume for the players consisted of 6.2 ± 2.1 h/week, which included integrated conditioning exercises in addition to weekend matches. All players and coaches from the different categories were previously informed, and informed parental consent and player assent were obtained. The procedures followed the rules established by the Declaration of Helsinki. The present research was approved by the Research Ethics Committee of the University of Huelva.

### 2.2. Measurements

A multidimensional test battery was used, including general as well as handball-specific tests. The tests consisted of body composition and anthropometrical determination, and physical fitness tests were included as part of their respective training periodization. All measurements were taken in the Sports Science Lab located in the Department of Sport Sciences at the University of Huelva. Physical assessment consisted of a lower and upper limp strength test, speed-agility tests, and a cardiorespiratory endurance test for each participant. All players were scheduled over three consecutive training days to take the different measurements without negative inferences between the performance tests. All of the subjects from the same handball team were called to perform the tests at the same time and on the same day. The measurement protocol schedule was: Monday, body composition and anthropometric determination and 30-m shuttle run test; Wednesday, dynamometry tests and Bosco test; and Thursday, agility T-test and Yo-Yo intermittent recovery test level 1.

### 2.3. Body Composition and Anthropometric Measurements

Height was determined with a stadiometer (0.1 cm, Secca 220, Spain). While this was done, players remained static in an anatomical position with their heels together. The highest point of their head was taken as a reference, with their hair remaining compressed. Body composition measurements were taken by multi-frequency bioelectrical impedance (Inbody 230 Multi-frequency Segmental Body Composition Analyzer, Barcelona, Spain). Subjects were placed barefoot in an anatomical position with their hands and feet on the electrodes, wearing light clothes and without metallic accessories (0.1 kg, Inbody 230, Barcelona, Spain). This position was maintained until the evaluator finished the test. The analyzer complied with the applicable European standards (93/42EEC, 90/384EEC) for use in the medical industry. The impedance between the body segments was measured while an alternating current (50 kHz, 90 mA) passed through the lower and upper body. Participants were informed in advance of the conditions that had to be sustained prior to measurement: no alcohol for at least 24 h before the measurement, no vigorous exercise for at least 12 h prior, no food or drink for at least 3 h prior, and urination immediately before the measurement. All measurements took place at the same time. The following measurements were taken: weight, body mass index, and lean mass and fat mass expressed as a percentage of total fat in the body. Arm span (0.1 cm) was assessed using a measuring tape (the distance between the tips of the longest fingers on each hand when both arms are extended laterally and maximally to shoulder height, with the subject’s back against the wall and palms facing forwards). The same researcher took all anthropometric measurements during the anthropometrical determination.

### 2.4. Physical–Physiological Profile Determination

The physical fitness measurements consisted of specific tests assessing lower and upper limb strength, speed and agility, and cardiorespiratory fitness [9,13,16,17].

Strength measurements. To assess upper limb strength, a handgrip strength test was performed bilaterally using a TKK5101 Grip D-class III dynamometer (Takey, Tokyo, Japan), accurate to 0.1 kg and complying with standardization standards [18]. Each participant performed the test twice with each hand (right and left, alternatively), with 1 min of rest between trials. Participants were instructed to gradually and continuously squeeze for at least 2 s, standing throughout the whole test, keeping their arm straight and avoiding touching the dynamometer with any part of their body, with the exception of the hand being measured. Subjects were encouraged to do their best when performing the tests.

To assess the strength of the lower limbs, the Bosco test was performed, evaluating three different jumps: squat jump (SJ), countermovement jump (CMJ), and Abalakov jump (ABK) (ChronoJump, Boscosystem®, Barcelona, Spain) [19]. Prior to testing, all participants underwent a standardized 5-min warm-up consisting of submaximal plyometric and skipping exercises. Participants were familiarized with the test procedures and performed three jump trials for each jump type. Afterwards, two maximal jumps of each type were recorded with 30 s of rest between them. The attempt in which the greatest jump length or height was obtained was taken for further analysis. For the SJ, the player adopted a static position with a maintained knee flexion of 90° from which they ascended vertically without any type of countermovement, making a maximum vertical jump. The hands had to be placed on the hips and kept in this position during the whole jump. For the CMJ, players started from standing position and were instructed to begin the jump with a downward movement which was immediately followed by a concentric upward movement, resulting in a maximal vertical jump. The hands had to be placed on the hips and kept in this position during the whole jump. For the ABK, the same procedure as for the CMJ was applied, but this test was performed with a full arm swing. Strong verbal encouragement was provided during all tests to motivate participants to make maximal effort.

### 2.5. Speed and Coordination Measurements

Speed and coordination characteristics were evaluated by a 30-m shuttle run and agility T-test, respectively (ChronoJump, Boscosystem®, Barcelona, Spain). For the test, three photoelectric cells were located at 0, 5, and 30 m that recorded the player’s path at these determined distances. Acceleration speed and displacement speeds were measured. Acceleration was determined by the average speed between the starting cell and the first cell at 5 m. Displacement speed was determined by the average speed between starting point cell and the 30 m cell. Each player started from a standing position, with the front foot 0.2 m from the first photocell beam. Two separate attempts were performed with a recovery time of 120 s between them. The best attempt was used for the analysis.

Handball agility/coordination was tested with a previously validated handball-specific modified version of the T-test [20]. The modified T-test combined short forward and backward lateral movements over a total distance of 20 m, simulating defensive movements that are characteristic in the sport of handball. Before testing, subjects completed a 5-min warm-up, including jogging and lateral displacements. All subjects performed each test with at least 3 min of rest between trials to ensure adequate recovery. Two photoelectric cells were placed at the starting line and at the finish line. The best attempt was used for the analysis.

### 2.6. Endurance Performance Measurements

To assess intermittent endurance performance, the players completed the Yo-Yo intermittent recovery test level 1 (Yo-Yo IR1) [21]. The Yo-Yo IR1 consists of repeated 2 x 20-m runs back and forth between the starting, turning, and finishing lines at a progressively increased speed controlled by audio bleeps from a tape recorder. Between each run, the subjects have a 10-s active rest period consisting of 2 x 5 m of jogging. When the subjects failed to reach the finishing line in time twice, the distance covered was recorded and represented the test result. Before the test, all subjects completed a warm-up period consisting of the first four runs in the test. All subjects were familiarized with the test using at least one pre-test. This test has been shown to have a high reproducibility for athletes in intermittent sports (coefficient of variation = 5%) [21].

### 2.7. Statistics

Data were analyzed using SPSS 25.0 for Windows (SPSS Inc. Chicago, IL, USA) and are expressed as mean values and standard deviation for the descriptive analysis. The standardizing of the variables was carried out using the Shapiro–Wilk test with Lilliefors correction. Homoscedasticity was determined with the Levene test. A one-way ANOVA test was performed to compare the differences across the three handball age categories. The effect sizes between categories were calculated using Cohen’s d, and the effect sizes were interpreted as small (d = 0.2 and 0.5), moderate (d = 0.5 and 0.8), and large (d > 0.8), with effect sizes inferior to 0.2 considered trivial [22]. The level of significance was set at 0.05. The independence among physical–physiological anthropometrical characteristics (upper and lower limb strength, motor and cardiorespiratory ability) by category was verified with Pearson correlations, and variance inflation factors were explored to identify evidence of multicollinearity. Simple mediation analyses were performed by age category using the macro PROCESS developed by Hayes [23] with a bootstrap threshold of 10,000 and model 4. If zero was not included in the 95% confidence interval (CI) of the estimate, we concluded that the indirect effect (IE) was statistically significant. In this way, we confirmed whether the category mediates the relationship between lean body mass and fat mass, both separately, and the different factors created in the physiological profile of the handball athletes. A total of five factors were created, each one being formed by the combination of the results obtained in different tests. Upper limb strength integrated the mean values from the bilateral handgrip. Lower limb strength was formed by combining the height for the three types of jumps (SJ, CMJ, and ABK). In terms of speed (s) and agility (s), the former was determined by the values from the 30-m test, while agility was established through the run time of the T-test for further analysis. Finally, VO_2_ max consisted of the results obtained in the Yo-Yo IR1. The standardized (β) and unstandardized (B) regression coefficients are presented for four equations: (a) the equation that regressed the mediator (age category) on the independent variable (lean mass or fat mass), (b) the equation that regressed the dependent variable (physical performance: upper limb strength, lower limb strength, velocity, agility T-test and VO_2_ max) and the mediator, (c) the equation that regressed the independent variable and the dependent variable, and finally (d) the equation that regressed the IE of the mediator (age category) on the relationship between the independent variable (lean mass or fat mass) and the dependent variable (physical performance).

## 3. Results

Table 1 shows the results of the one-way ANOVA analyses used to explore the differences between anthropometrical characteristics by category. No significant differences were observed according to category for the majority of the measured parameters, except for age, height, lean body mass, and arm span. Lean body mass increased significantly as player age category increased (*p* < 0.05; **∆** = 4.66–9.38; effect size (ES) = 0.96–1.92).

Table 2 shows the results of the comparative analysis for the physical–physiological profile according to category. With regard to upper and lower limb strength, handball players showed significant differences (*p* < 0.05) between the three categories in both the right and left handgrip, as well as the SJ, CMJ, and ABK. For handgrip strength, the U18 category showed the highest values (40.5 ± 6.69 and 35.9 ± 7.12 for right and left handgrip, respectively; ES = 1.04–1.97). In both the SJ and CMJ, significant differences were observed among all three categories in relation to jump height and power, with significant differences being found between U18 vs. U14 and U18 vs. U16. For the ABK, significant differences in jump height were found between the U18 vs. U14 and U18 vs. U16 categories. In contrast, significant differences were only found between U18 vs. U14 with respect to jumping power.

The speed and coordination characteristics of the handball players were assessed using the 30-m shuttle run and agility T-tests. In both speed and agility tests, players’ coordination and velocity improved as the category increased (*p* < 0.05). However, no significant differences were found between the U18 vs. U16 categories. More specifically, in the T-test, significant differences were obtained between the U18 vs. U14 and U18 vs. U16 categories for both the completion time (*p* < 0.05; **∆** = 0.31–0.52; ES = 1.27–2.24) and the speed (*p* < 0.05; **∆** = 1.08–1.72; ES = 1.28–2.11) reached by the subjects. Similarly, the results regarding the 30-m speed test showed that, as age increased, the speed reached by the players was greater (*p* < 0.05) for both the global test and for the test over 5-m and 25-m sections.

Finally, the Yo-Yo IR1 test was performed to determine the players’ aerobic capacity. The results of the test showed an increase in aerobic capacity as the age category increased. It is worth highlighting that there were significant differences between the U18 vs. U14 and U18 vs. U16 categories in all of the measured parameters.

Table 3 shows the matrix correlations for the relationship between anthropometrical characteristics and the physical–physiological profile by category. In relation to the bivariate correlation analysis for the U14 category, all anthropometric variables were positively correlated with jump power (SJ and CMJ) (r = 0.703–0.953; all *p* < 0.001). However, weight, BMI, and fat percentage were negatively associated with jump height (SJ, CMJ, and ABK) (r = −0.465 to −0.777; all *p* < 0.001). For the speed and agility/coordination tests, weight, BMI, and fat percentage were negatively associated with speed (r = −0.391 to −0.548; all *p* < 0.05) and positively associated with time. Likewise, weight, BMI, and fat percentage were negatively associated with the number of laps and VO_2_ max (r = −0.596 to −0.818; all *p* < 0.05). Regarding U16, height, lean mass, and arm span were positively correlated with handgrip in both hands (r = 0.590–0.815; *p* < 0.05). Similarly, body mass, BMI, and lean mass were positively correlated with jump height (SJ and CMJ) (r = 0.626–0.656; *p* < 0.001). For the speed test, fat percentage was negatively associated with speed and positively associated with time (r = 0.659; *p* < 0.05). Finally, in the U18 category, a positive correlation was found between right handgrip and weight, BMI, and lean mass (r = 0.597–0.673; *p* < 0.05). Moreover, CMJ power was positively correlated with BMI (r = 0.556; *p* < 0.05) and there was a negative correlation between fat mass and 30-m speed (r = −0.801; *p* < 0.05). The correlation analyses in all samples indicated that age was positively correlated with better scores for all physical–physiological parameters. Additionally, greater lean mass was positively correlated with better scores for all physical condition tests (r = 0.350–0.849; all *p* < 0.001). However, a higher fat percentage negatively influenced lower limb strength, speed, agility/coordination, and cardiorespiratory tests (r = –0.554 to –0.676; all *p* < 0.001).

**Figure 1:**
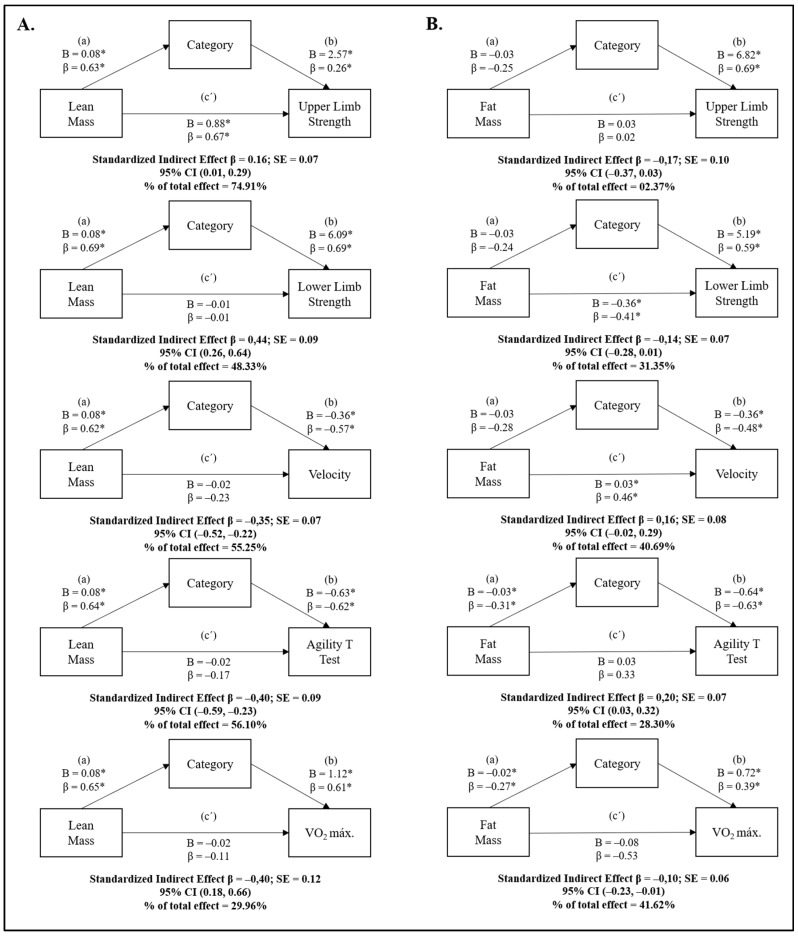
**A.** Simple mediation analysis of lean mass on the physiological profile of handball players in relation to category. **B.** Simple mediation analysis of fat mass on the physiological profile of handball players in relation to category. B: Unstandardized regression coefficients; β: Standardized regression coefficients; SE: Standard error; CI: Confidence interval. * statistically significant at *p* < 0.05.

Figure 1 illustrates the results of the bootstrapped mediation analysis to determine whether handball category mediates the relationship between lean mass or fat mass scores and the physical–physiological profile of youths. Regarding the mediation effect of category on lean mass and the physical–physiological profile, in the first regression equation (a), lean mass was positively linked to category (β = 0.63–0.69; *p* < 0.05). Moreover, the second equation (b) for category and the different physical–physiological dimensions showed a direct correlation with upper and lower limb strength (β = 0.23–0.69; *p* < 0.05) and cardiorespiratory fitness (β = 0.61; *p* < 0.05), and was negatively associated with time in the speed and agility tests (β = −0.57 to −0.62; *p* < 0.05). Additionally, the bootstrapped CIs for the indirect effect suggest that handball category mediated the relationship between lean mass and all physical capacities (Figure 1A), and 74.91%, 48.33%, 55.25%, 56.10%, and 29.96% of the total effect was accounted for by upper and lower limb strength, velocity, agility, and cardiorespiratory fitness, respectively. The category showed a greater indirect effect on the relationship between lean mass and lower body strength (standardized indirect effect β = 0.44; SE = 0.09; 95% CI = 0.26, 0.64). In relation to the effect the category had on fat mass and the physical–physiological profile, in the first regression equation (a), fat mass was negatively related to agility and cardiorespiratory fitness (β = −0.27 to −0.31; *p* < 0.05). The second equation (b) for handball category and physical capacities showed a direct correlation with upper and lower limb strength (β = 0.59–0.69; *p* < 0.05), as well as cardiorespiratory fitness (β = 0.39; *p* < 0.05), and was negatively related to time in the speed and agility tests (β = −0.48 to −0.63; *p* < 0.05). Finally, the bootstrapped CIs for the indirect effect suggest that handball category mediated the relationship between fat mass in both agility (standardized indirect effect β = 0.20; SE = 0.07; 95% CI = 0.03, 0.32) and cardiorespiratory capacity (standardized indirect effect β = −0.10; SE = 0.06; 95% CI = −0.23, −0.01) (Figure 1B), and 28.30% and 41.62% of the total effect was accounted for agility and cardiorespiratory fitness, respectively.

## 4. Discussion

In recent years, studies have noted the importance of assessing the physical performance of young handball players from different countries and the influence of age, maturity status, and anthropometric characteristics upon their performance [6,7,8,9,10,13,24]; however, the study of lean mass and its direct influence on physical performance is limited to date. To our knowledge, there are no studies that evaluate the mediating effect of body composition through lean mass and fat mass on the different physical qualities that a handball player requires. The present study initially aimed to determine the differences in body composition through lean body mass and fat mass and the physical and physiological profile of handball youths across age categories. One of our main findings showed a progressive increase in all body composition parameters, mainly influenced by age, except for lean mass, which remained statistically significant when age-adjusted and compared by category. Additionally, the comparative analysis of physical performance between categories showed an improvement in strength, speed, agility, and cardiorespiratory capacity as the category increased, especially when comparing U14 with U16 and U18. These differences were reduced or not appreciable for speed, agility, or cardiorespiratory fitness when comparing categories U16 and U18. As a novelty, the present study examined the indirect effect of category mediation on the relationship between lean mass or fat mass and the physical fitness profile of handball youths. It is important to highlight that the category had an indirect mediating effect on the relationship between lean mass and physical performance measured through upper and lower body strength, speed, agility, and cardio-respiratory fitness. In contrast, in relation to fat mass and its effect on different physical capacities, the only mediating effects observed for the category were for agility and cardio-respiratory capacity. Therefore, these results demonstrate a greater usefulness of the analysis of lean mass even over fat mass, given the greater association with physical performance.

The importance in determining body composition is a key factor in the characterization of a specific anthropometrical profile in both young and adult handball athletes. Several studies have paid special attention to determining their relationship based on age or biological maturity [7,8,9], specific positions [12], and game level in handball youths [13]. In a similar population, Matthys et al. [9] evaluated handball youths from the U14, U15, and U18 categories, distinguishing them according to elite and non-elite levels. Our handball youths showed similar results to their non-elite players in anthropometrical parameters such as height, weight, fat percentage, and arm span, with our results being slightly below their elite youth [13,16]. Specifically, they found a direct relationship between biological maturation and all anthropometrical characteristics except body fat, and two more studies [25,26] verified this relative age-dependent effect in young elite handball athletes. In our study, when analyzing body composition by categories and after age adjustment, we observed that many of the differences disappeared, except for lean mass. This would mean that age is not the only determining factor in these players’ body composition, but that category demands may also have an impact on body composition, although less than the age-promoted effects. In accordance with this, a recent study [6] observed that marked differences in both anthropometric characteristics and physical performance between age categories were described by calendar age, suggesting that it may reflect not only the impact of physical growth, but also the accumulation of training, technique, and playing experience.

Handball players should be able to perform repeated high-intensity actions; therefore, a well-developed physical–physiological profile is considered a determinant for handball players. We observed that our players exhibited slightly lower values for all tests performed, except the 30-m sprint test, where our results slightly exceeded those obtained by other studies in similar groups [9,13,16,17]. Interestingly, we reported similar upper limb strength in our U18 category to that reported by Vila Suárez et al. [17] in Spanish handball youths from the same category. With respect to lower limb strength, our CMJ and SJ values were similar to the those reported by Ingebrigtsen et al. [16] in both the U16 and U18 categories, and the ABK value in U14 athletes reported by Camacho-Cardenosa et al. [10]. As for cardiorespiratory capacity, the values obtained by Camacho-Cardenosa et al. [10] and Rousanoglou et al. [12] were higher in the three analyzed categories. Surprisingly, we observed clear differences for VO_2_ max comparison, especially in our U18 category, although the last study mentioned used the 20-m shuttle run test without considering the intermittent activity that handball presents, so its values may have been overestimated. Finally, the results obtained in the T-test revealed a better score compared to lower categories; however, no significant differences were observed between the two upper categories. This confirms that young players do not necessarily increase differences with regard to their physical performance as their specialization stage progresses, so that the highest profits would be at the beginning of the specialization stage, and as specialization progresses, these differences would be reduced between categories. In this line, it has been described that the morphological characteristics also tend to stabilize at the age of 16–17 years, at least in European children [27].

In handball, young athletes perform better according to their anthropometrics and physiological characteristics [13,26]. Although there is an age influence [28], we have confirmed that greater significant differences in body composition and physical fitness were observed when comparing U14 to the U16 or U18 categories; however, these differences disappeared when comparing U16 to U18, especially in agility/coordination, speed, or cardiorespiratory capacity. To the best of our knowledge, the last two published studies on this topic [6,7] still considered anthropometric parameters such as weight, height, BMI, or fat percentage to demonstrate the influence of anthropometrical parameters on physical performance. However, a small number of studies have focused on the potential importance of accessing lean mass in a similar collective [6,12]. While in lower categories, fat percentage could be useful due to our univariate associations between fat mass and physical performance in the U14 category (Table 3), this compartment clearly becomes less relevant in higher categories. Even though the scientific literature has shown a clear relationship between some general body composition parameters like height, weight, or BMI and physical qualities such as throwing speed or jump strength capacity [29,30], no studies have analyzed the mediation effect of body compartments that could be as relevant for performance as both lean mass or fat mass on the physiological profile by category in young handball players. Interestingly, our study demonstrates that category plays a role as a mediator between lean mass or fat mass and the different physiological capacities in youth handball athletes. In our study, the indirect effect suggests that handball category mediated the relationship between lean mass and all physical capacities, and 74.91%, 48.33%, 55.25%, 56.10%, and 29.96% of the total effect was accounted for by upper and lower limb strength, velocity, agility, and cardiorespiratory fitness, respectively, indicating that this compartment is more relevant even than fat mass. Additionally, fat content showed a mediating effect on important qualities such as agility/coordination and cardiorespiratory fitness. This would mean that as the category increases, this would adversely affect both capabilities. Studies such as that of Granados [31] observed that greater fat-free mass was linked to a better performance, mainly due to the direct relationship with muscular power and strength. Confirming this, Ortega-Becerra et al. [7] found that handball youths showed greater throwing velocities, jump height, running sprint capacity, and muscle strength in their upper and lower extremities as their handball performance increased [7], which emphasizes the importance of evaluating lean mass in these young players.

For future research, it would be interesting to explore the evolution of key body compartments such as lean body mass in conjunction with age or degree of maturity, category, and specific position in the athletes’ performance. In this respect, recent studies [6] have indicated that future research should examine the impact of other anthropometric characteristics, such as chest circumference and the length and volume of the upper limbs. This would be valuable information for coaches, highlighting the need to control, as well as promote, specific strategies for an increase in lean mass in young players. As limitations of this study, taking this research as a reference, it would be interesting for future research to consider a larger sample size within a single category in order to increase the representativeness of the research, and to conduct a longitudinal study to verify how body composition parameters progress and how physical condition is modulated. Other important limitations include the inability to examine the influence of maturity status, playing position, or competitive level. On the other hand, our upper limb strength integrated the mean values from the bilateral handgrip strength capacity; however, a more precise model could also have comprised the muscles involving the shoulders and elbows. Further, a multiple mediation analysis could also be performed where two variables are considered to be mediators. Moreover, it would be interesting to analyze the results obtained over recent years in handball youths to establish possible reference values regarding the physical qualities that determine handball performance according to age category.

## 5. Conclusions

To conclude, the results of the present study showed a progressive increase in all body composition parameters, mainly influenced by age, except for lean mass, as well as an improvement in strength, speed, agility, and cardiorespiratory capacity as the category increased. Handball age category presented an indirect mediating effect between lean mass and physical performance measured through upper and lower body strength, speed, agility, and cardio-respiratory fitness. Therefore, these results demonstrate a greater usefulness of the analysis of lean mass over even fat mass, given the greater association with physical performance.

## Figures and Tables

**Table 1:** Sample characteristics.

Characteristics	U14		U16		U18		∆ U16 vs. U14	∆ U18 vs. U14	∆ U18 vs. U16	ES U16 vs. U14	ES U18 vs. U14	ES U18 vs. U16
	Mean	SD	Mean	SD	Mean	SD						
Age (years)	13.7	0.48	15.1	0.32	17.1	0.57	1.42 ^b^	3.37 ^b^	1.97 ^b^	3.43	6.50	4.40
Height (m)	1.61	0.09	1.72	0.05	1.75	0.07	0.11 ^b^	0.13 ^b^	0.02	1.14	1.72	0.46
Body mass (kg)	60.6	15.5	63.5	10.6	70.0	11.2	2.93	9.34	6.40	0.22	0.69	0.60
BMI (kg·m2-1)	23.0	4.52	21.3	3.05	22.8	3.01	−1.66	−0.20	1.45	−0.44	−0.05	0.50
Lean mass (kg)	24.6	4.74	29.3	4.78	34.0	5.08	4.71 ^a^	9.38 ^b^	4.66 ^a^	0.99	1.92	0.96
Fat mass (%)	16.2	9.81	11.2	8.71	10.1	5.29	−5.00	−6.04	−1.03	0.54	0.76	0.15
Arm span (cm)	154.0	38.6	174.9	9.04	173.2	8.14	20.8 ^a^	19.1	−1.72	0.74	0.66	0.21

Data are mean ± standard deviation (SD). U14, between 13 and 14 years (*n* = 19); U16, between 15 and 16 years (*n* = 18); U18, between 17 and 18 years (*n* = 16). BMI: Body mass index; ES: Effect size. Statistically significant differences by category: a = *p* < 0.05; b = *p* < 0.001.

**Table 2:** Comparative analysis of the physiological profile of the players by category.

	U14		U16		U18		∆ U16 vs. U14	∆ U18 vs. U14	∆ U18 vs. U16	ES U16 vs. U14	ES U18 vs. U14	ES U18 vs. U16
	Mean	SD	Mean	SD	Mean	SD						
**Upper limb strength**												
Handgrip Right (kg)	27.4	6.60	34.3	5.41	40.5	6.69	6.91 ^b^	13.0 ^c^	6.13 ^a^	1.14	1.97	1.04
Handgrip Left (kg)	25.1	6.17	31.0	6.19	35.9	7.12	5.91 ^a^	10.8 ^c^	4.86	0.96	1.64	0.74
**Lower limb strength**												
SJ Height (cm)	21.3	5.36	26.4	4.28	32.8	6.92	5.10 ^a^	11.5 ^c^	6.37 ^b^	1.04	1.89	1.12
SJ Power (W)	1888.5	514.2	2281.0	667.1	2994.8	734.1	392.5	1106.3 ^c^	713.8 ^a^	0.65	1.75	1.02
CMJ Height (cm)	24.0	5.29	29.0	4.75	34.8	6.13	4.97 ^a^	10.9 ^c^	5.90 ^a^	0.99	1.90	1.06
CMJ Power (W)	2066.9	591.5	2467.7	657.0	3109.1	674.3	400.8	1042.1 ^c^	641.3 ^a^	0.64	1.64	0.96
ABK Height (cm)	28.5	5.35	32.2	5.50	40.0	5.72	3.71	11.5 ^c^	7.78 ^c^	0.68	2.08	1.39
ABK Power (W)	1891.1	1134.7	2270.0	1324.7	3062.8	1334.7	378.9	1171.7 ^a^	792.8	0.68	2.09	1.38
**Agility/coordination**												
Time (s)	10.19	0.95	9.11	0.74	8.47	0.52	1.08 ^a^	1.72 ^a^	0.64	1.28	2.11	0.95
Velocity (m·s-1)	2.64	0.25	2.94	0.24	2.87	0.97	0.31 ^a^	0.52 ^a^	0.21	1.27	2.24	0.93
**Velocity**												
Total Time 30 m (s)	5.20	0.39	4.52	0.40	4.35	0.18	−0.63 ^c^	−0.80 ^c^	−0.17	1.60	2.50	0.54
Total Velocity 30 m (m·s-1)	5.9	0.45	6.69	0.64	6.91	0.31	0.83 ^c^	1.05 ^c^	0.22	1.53	2.64	0.43
Time 5 m (s)	1.20	0.16	1.06	0.08	1.06	0.07	−0.15 ^c^	−0.15 ^b^	0.00	1.15	1.15	0.00
Velocity 5 m (m·s-1)	4.2	0.47	4.75	0.36	4.74	0.31	0.56 ^c^	0.55 ^c^	−0.01	1.32	1.34	0.03
Time 25 m (s)	3.9	0.44	3.46	0.39	3.29	0.15	−0.42 ^b^	−0.59 ^c^	−0.17	1.00	0.73	0.20
Velocity 25 m (m·s-1)	6.5	0.89	7.32	1.03	7.61	0.35	0.79 ^a^	1.08 ^b^	0.29	0.83	1.51	0.37
**Cardiorespiratory measures**												
Laps (nº)	13.7	1.00	15.2	1.32	15.6	1.33	1.51 ^b^	1.95 ^c^	0.45	1.27	1.63	0.33
VO_2_ max (ml·min·kg-1)	40.4	1.60	43.5	3.29	44.9	3.01	3.13 ^a^	4.50 ^b^	1.37	1.17	1.91	0.44

Data are mean ± SD. U14, between 13 and 14 years (*n* = 19); U16, between 15 and 16 years (*n* = 18); U18, between 17 and 18 years (*n* = 16). ES: Effect size; SJ: Squat jump; CMJ: Countermovement jump; ABK: Abalakov jump. Statistically significant differences by category: a = *p* < 0.05; b = *p* < 0.01; c = *p* < 0.001.

**Table 3:** Matrix for correlation coefficients (r) showing the simple linear relationship between anthropometrical characteristics and the physical–physiological profile by category.

	Handgrip Right (kg)	Handgrip Left (kg)	SJ Height (cm)	SJ Power(W)	CMJ Height (cm)	CMJ Power (W)	ABK Height (cm)	T-Test Time (s)	T-Test Velocity (m·s-1)	30 m Time (s)	30 m Velocity (m·s-1)	Yo-Yo IR1 Laps (nº)	VO_2_ max (ml·min·kg-1)
U14													
Height (m)	0.642 ^b^	0.563 ^b^	−0.091	0.839 ^b^	−0.110	0.832 ^b^	0.074	0.099	−0.072	−0.147	0.169	−0.318	−0.207
Body mass (kg)	0.367	0.276	−0.585 ^b^	0.927 ^b^	−0.527 ^b^	0.953 ^b^	−0.465 ^b^	0.376 ^a^	−0.391 ^a^	0.405 ^a^	−0.396 ^a^	−0.663 ^b^	−0.596 ^b^
BMI (kg·m2-1)	0.129	0.062	−0.684 ^b^	0.732 ^b^	−0.616 ^b^	0.782 ^b^	−0.612 ^b^	0.416 ^a^	−0.441 ^a^	0.592 ^b^	−0.599 ^b^	−0.718 ^b^	−0.703 ^b^
Lean mass (kg)	0.749 ^b^	0.630 ^b^	−0.175	0.914 ^b^	−0.085	0.914 ^b^	0.019	0.035	−0.036	−0.115	0.136	−0.307	−0.231
Fat mass (%)	−0.026	−0.076	−0.777 ^b^	0.703 ^b^	−0.767 ^b^	0.744 ^b^	−0.748 ^b^	0.526 ^b^	−0.548 ^b^	0.697 ^b^	−0.701 ^b^	−0.818 ^b^	−0.775 ^b^
Arm span (cm)	0.032	0.086	−0.036	0.805 ^b^	−0.156	0.798 ^b^	−0.043	0.248	−0.230	0.132	−0.137	−0.237	−0.137
U16													
Height (m)	0.784 ^b^	0.590 ^a^	−0.024	0.387	−0.080	0.388	−0.066	−0.411	0.411	−0.069	0.026	0.198	0.235
Body mass (kg)	0.506	0.590 ^a^	−0.259	0.704 ^b^	−0.364	0.731 ^b^	−0.259	0.003	−0.005	0.154	−0.136	−0.363	−0.297
BMI (kg·m2-1)	0.212	0.392	−0.288	0.626 ^b^	−0.376	0.656 ^b^	−0.277	0.190	−0.191	0.246	−0.194	−0.490	−0.433
Lean mass (kg)	0.802 ^b^	0.815 ^b^	0.143	0.576 ^a^	0.040	0.574 ^a^	0.238	−0.364	0.369	−0.283	0.240	0.070	0.126
Fat mass (%)	−0.065	0.075	−0.393	0.403	−0.447	0.432	−0.479	0.314	−0.323	0.659 ^a^	−0.571 ^a^	−0.466	−0.435
Arm span (cm)	0.777 ^b^	0.609 ^a^	−0.020	0.520	−0.175	0.500	−0.064	−0.421	0.428	0.001	−0.031	0.093	0.154
U18													
Height (m)	0.359	0.293	−0.027	0.226	0.016	0.314	0.084	−0.769 ^b^	0.777 ^b^	−0.022	0.004	0.293	0.407
Body mass (kg)	0.410	0.640 ^a^	−0.239	0.433	−0.288	0.556 ^a^	−0.275	−0.259	0.238	0.411	−0.404	−0.260	−0.131
BMI (kg·m2-1)	0.302	0.597 ^a^	−0.319	0.386	−0.399	0.486	−0.418	0.193	−0.220	0.550	−0.530	−0.484	−0.391
Lean mass (kg)	0.472	0.673 ^a^	−0.003	0.417	−0.043	0.512	−0.030	−0.521	0.498	−0.021	0.017	−0.022	0.102
Fat mass (%)	0.153	0.329	−0.469	0.293	−0.514	0.400	−0.524	0.266	−0.274	0.821 ^b^	−0.801 ^b^	−0.528	−0.449
Arm span (cm)	0.247	0.247	−0.132	0.490	−0.116	0.604	−0.074	−0.601	0.606	0.055	−0.059	0.113	0.237
ALL													
Height (m)	0.763 ^b^	0.697 ^b^	0.420 ^b^	0.720 ^b^	0.410 ^b^	0.742 ^b^	0.477 ^b^	−0.488 ^b^	0.541 ^b^	−0.539 ^b^	0.545 ^b^	0.367 ^b^	0.451 ^b^
Body mass (kg)	0.493 ^b^	0.507 ^b^	−0.012	0.758 ^b^	−0.027	0.821 ^b^	0.025	−0.046	0.092	0.006	0.031	−0.184	−0.067
BMI (kg·m2-1)	0.087	0.133	−0.339 ^b^	0.457 ^b^	−0.358 ^b^	0.532 ^b^	−0.332 ^b^	0.339 ^a^	−0.320 ^a^	0.411 ^b^	−0.368 ^b^	−0.524 ^b^	−0.430 ^b^
Lean mass (kg)	0.849 ^b^	0.837 ^b^	0.483 ^b^	0.814 ^b^	0.484 ^b^	0.829 ^b^	0.547 ^b^	−0.570 ^b^	0.618 ^b^	−0.585 ^b^	0.598 ^b^	0.350 ^b^	0.430 ^b^
Fat mass (%)	−0.260	−0.232	−0.587 ^b^	0.201	−0.617 ^b^	0.278 ^a^	−0.625 ^b^	0.564 ^b^	−0.554 ^b^	0.710 ^b^	−0.673 ^b^	−0.676 ^b^	−0.587 ^b^
Arm span (cm)	0.329 ^a^	0.345 ^b^	0.225	0.719 ^b^	0.166	0.744 ^b^	0.208	−0.189	0.232	−0.204	0.217	0.301 ^a^	0.383 ^b^

SJ: Squat jump; CMJ: Countermovement jump; ABK: Abalakov jump; BMI: body mass index. Matrix correlations are presented as correlation coefficients (r). a = statistically significant at *p* < 0.05; b = statistically significant at *p* < 0.001.

## References

[1] Massuça L.M., Fragoso I., Teles J. (2014). Attributes of Top Elite Team-handball Players. J. Strength Cond. Res..

[2] Hermassi S., Chelly M.-S., Wollny R., Hoffmeyer B., Fieseler G., Schulze S., Irlenbusch L., Delank K.-S., Shephard R.J., Bartels T. (2018). Relationships between the handball-specific complex test, non-specific field tests and the match performance score in elite professional handball players. J. Sports Med. Phys. Fit..

[3] Michalsik L.B., Aagaard P., Madsen K. (2013). Locomotion characteristics and match-induced impairments in physical performance in male elite team handball players. Int. J. Sports Med..

[4] Michalsik L.B., Madsen K., Aagaard P. (2015). Technical match characteristics and influence of body anthropometry on playing performance in male elite team handball. J. Strength Cond. Res..

[5] Ronglan L.T., Raastad T., Børgesen A. Neuromuscular Fatigue and Recovery in Elite Female Handball Players. https://onlinelibrary.wiley.com/doi/abs/10.1111/j.1600-0838.2005.00474.x.

[6] Hammami M., Hermassi S., Gaamouri N., Aloui G., Comfort P., Shephard R.J., Chelly M.S. (2019). Field Tests of Performance and Their Relationship to Age and Anthropometric Parameters in Adolescent Handball Players. Front. Physiol..

[7] Ortega-Becerra M., Pareja-Blanco F., Jiménez-Reyes P., Cuadrado-Peñafiel V., González-Badillo J. (2018). Determinant Factors of Physical Performance and Specific Throwing in Handball Players of Different Ages. J. Strength Cond. Res..

[8] Hammami R., Sekulic D., Selmi M., Fadhloun M., Spasic M., Uljevic O., Chaouachi A. (2018). Maturity Status as a Determinant of the Relationships Between Conditioning Qualities and Preplanned Agility in Young Handball Athletes. J. Strength Cond. Res..

[9] Matthys S.P.J., Fransen J., Vaeyens R., Lenoir M., Philippaerts R. (2013). Differences in biological maturation, anthropometry and physical performance between playing positions in youth team handball. J. Sports Sci..

[10] Camacho-Cardenosa A., Camacho-Cardenosa M., González-Custodio A., Martínez-Guardado I., Timón R., Olcina G., Brazo-Sayavera J. (2018). Anthropometric and Physical Performance of Youth Handball Players: The Role of the Relative Age. Sports.

[11] Hermassi S., Laudner K., Schwesig R. (2019). Playing Level and Position Differences in Body Characteristics and Physical Fitness Performance among Male Team Handball Players. Front. Bioeng. Biotechnol..

[12] Rousanoglou E.N., Noutsos K.S., Bayios I.A. (2014). Playing level and playing position differences of anthropometric and physical fitness characteristics in elite junior handball players. J. Sports Med. Phys. Fit..

[13] Matthys S.P.J., Vaeyens R., Fransen J., Deprez D., Pion J., Vandendriessche J., Vandorpe B., Lenoir M., Philippaerts R. (2013). A longitudinal study of multidimensional performance characteristics related to physical capacities in youth handball. J. Sports Sci..

[14] Ziv G., Lidor R. (2009). Physical characteristics, physiological attributes, and on-court performances of handball players: A review. Eur. J. Sport Sci..

[15] Tovar-Galvez M.I., González-Jiménez E., Martí-García C., Schmidt-RioValle J. (2017). Body composition in a population of school adolescents: A comparison of simple anthropometric methods and bioelectrical impedance. Endocrinol. Diabetes Nutr..

[16] Ingebrigtsen J., Jeffreys I., Rodahl S. (2013). Physical Characteristics and Abilities of Junior Elite Male and Female Handball Players. J. Strength Cond. Res. Natl. Strength Cond. Assoc..

[17] Suárez H.V. (2009). Estudio del perfil antropométrico del jugador juvenil de balonmano en la Región de Murcia. RETOS Nuevas Tend. Educ. Física Deporte Recreación.

[18] España-Romero V., Ortega F.B., Vicente-Rodríguez G., Artero E.G., Rey J.P., Ruiz J.R. (2010). Elbow Position Affects Handgrip Strength in Adolescents: Validity and Reliability of Jamar, DynEx, and TKK Dynamometers. J. Strength Cond. Res..

[19] Pueo B., Jimenez-Olmedo J.M., Lipińska P., Buśko K., Penichet-Tomas A. (2018). Concurrent validity and reliability of proprietary and open-source jump mat systems for the assessment of vertical jumps in sport sciences. Acta Bioeng. Biomech..

[20] Sassi R.H., Dardouri W., Yahmed M.H., Gmada N., Mahfoudhi M.E., Gharbi Z. (2009). Relative and absolute reliability of a modified agility T-test and its relationship with vertical jump and straight sprint. J. Strength Cond. Res..

[21] Krustrup P., Mohr M., Amstrup T., Rysgaard T., Johansen J., Steensberg A., Pedersen P.K., Bangsbo J. (2003). The yo-yo intermittent recovery test: Physiological response, reliability, and validity. Med. Sci. Sports Exerc..

[22] Cohen J. (1988). Statistical Power Analysis for the Behavioral-Sciences.

[23] Hayes A.F. (2013). Introduction to Mediation, Moderation, and Conditional Process Analysis: A Regression-Based Approach.

[24] Prieske O., Chaabene H., Puta C., Behm D.G., Büsch D., Granacher U. (2019). Effects of Drop Height on Jump Performance in Male and Female Elite Adolescent Handball Players. Int. J. Sports Physiol. Perform..

[25] Fonseca F.S., Figueiredo L.S., Gantois P., de Lima-Junior D., Fortes L.S. (2019). Relative Age Effect is Modulated by Playing Position but is Not Related to Competitive Success in Elite Under-19 Handball Athletes. Sports.

[26] Nikolaidis P.T., Ingebrigtsen J., Povoas S.C., Moss S., Torres-Luque G. (2015). Physical and physiological characteristics in male team handball players by playing position—Does age matter?. J. Sports Med. Phys. Fit..

[27] Van Praagh E., Doré E. (2002). Short-term muscle power during growth and maturation. Sports Med. Auckl. NZ.

[28] Malina R., Rogol A., Cumming S., Coelho-e-Silva M., Figueiredo A. (2015). Biological maturation of youth athletes: Assessment and implications. Br. J. Sports Med..

[29] Debanne T., Laffaye G. (2011). Predicting the throwing velocity of the ball in handball with anthropometric variables and isotonic tests. J. Sports Sci..

[30] Schwesig R., Hermassi S., Fieseler G., Irlenbusch L., Noack F., Delank K.-S., Shephard R.J., Chelly M.-S. (2017). Anthropometric and physical performance characteristics of professional handball players: Influence of playing position. J. Sports Med. Phys. Fit..

[31] Granados C., Izquierdo M., Ibañez J., Bonnabau H., Gorostiaga E.M. (2007). Differences in physical fitness and throwing velocity among elite and amateur female handball players. Int. J. Sports Med..

